# Why Feminist Participatory Methods Matter for Global Health Research in Sub‐Saharan Africa

**DOI:** 10.1002/gch2.202500066

**Published:** 2026-01-08

**Authors:** Heather M. Tucker, Don Catherine Awuor Ochieng

**Affiliations:** ^1^ Center For AIDS Prevention University of California, San Francisco San Francisco California USA; ^2^ Women Working with Women (3 W) Kisumu Kenya

**Keywords:** feminist, global health, methods, participatory, power

## Abstract

Research in global health is often framed as centering health equity. However, research and programmatic partnerships are often relationships between institutions and researchers in high‐income countries (HICs), and researchers and actors in low‐ and middle‐income countries (LMICs), including many countries in Sub‐Saharan Africa (SSA). Such relationships are rife with power dynamics that require thoughtful attention and solutions. Feminist research methods, including perspectives from intersectional and African feminist thinkers, as well as participatory approaches, may offer a means of engaging with power inequities and disrupting often taken‐for‐granted assumptions in the SSA context. Such epistemological perspectives and methods not only disrupt “traditional” research relationships and challenge unexamined assumptions about knowledge but are also driven by the lived experiences of health disparities in specific, formerly colonized contexts and can therefore lead to context‐specific or localized solutions to complex health inequities. This paper explores how these specific feminist research perspectives and methods can challenge power dynamics that are often embedded in global health research and interventions in SSA.

## Graphical abstract

Research in global health is often framed as centering health equity. Research and programmatic partnerships are often relationships that are rife with power inequities. Feminist research methods, including perspectives from intersectional and African feminist thinkers, as well as participatory approaches, may offer a means of engaging with power inequities and disrupting global health inequities that are common in the sub‐Saharan African context, specifically.

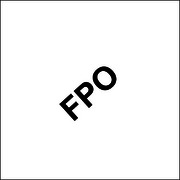

## 1 Introduction

The global health research process and relationship between institutions, organizations, researchers/collaborators is often a transnational collaboration between actors in the global north or high‐income countries (HICs) with those in the global south or low to middle‐income countries (LMICs), including many countries in Sub‐Saharan Africa (SSA). These transnational collaborations, however, are embedded in unequal systems of power that require careful and methodological attention. Critical global health scholarship proposing to “decolonize” global health has articulated how global health as a field of study, industry, and philosophy is rooted in racialized, colonial, and missionary legacies that are often rife with assumptions and practices that remain neocolonial [1, 2].

In fact, scholars have highlighted the need to critically consider that global health has grown from a history of “tropical medicine” in colonial projects in SSA, which often relied on paternalistic approaches, scientific racism, and targeted local populations with health campaigns and legislation to control behaviors [2, 3, 4]. Banerjee et al (2023) have articulated how the colonial perspective embedded in current global health educational and research systems can intend to work on social justice issues, but rely on a “white saviour complex,” perpetuating inequities and removing agency and context from partners’ experiences [5]. Global health research in SSA can reflect colonial power dynamics, with researchers from HICs framing agendas focused on vulnerable populations that are often viewed as victims, diseased, and traumatized, with little room for complexity, agency, pleasure, and nuance [6, 7]. From these perspectives, global health researchers are all too often influenced by the pressures and visions of donors in HICs to create an impact in SSA. However, this often ignores former violent colonial histories and the introduction of neoliberal market logics and donor reliance, which have positioned many SSA countries in a mode of dependence on HIC financial institutions and countries for resources such as healthcare [8]. Furthermore, with global health research funding located in institutions in the global north, often the power to determine the scope, methods, and limitations of research that takes place in SSA is governed by researchers from HICs, disconnected from on the ground realities. This is inclusive of a Euro‐centric, medicalized epistemological approach to research, which draws from “hard” medical science for solutions to often complex, social health problems that are often connected to structural global inequities. This sole reliance on scientific methods, including biomedical science and positivist methods, has been identified by critical global health scholars as a form of neocolonialism, as these methods are held as superior to others, or as epistemological truths [4, 9, 10].

Feminist researchers have also critiqued these approaches to transnational research. This has included the need to pay attention to the ways in which this pressure to generate specific medical and health focused outcomes through positivist means, invisibilizes and devalues other methods or ways of knowing, and therefore, potentially loses information that can capture the complexity of experiential knowledge [11, 12]. As a way forward in challenging forms of neo‐colonialism in global health, scholars have called attention to the critical need for feminist perspectives, including intersectional perspectives and methodologies, in global health research and practice [9, 13, 14, 15, 16], and there have been calls to bring intersectional thinking into global health, specifically as a means to think through health disparities [13]. Eger et al (2024) have highlighted the need for a comprehensive framework for feminist global health policy, as an initial guide inclusive of “power regimes, intersectionality and knowledge paradigms,” but which must also be ‘context specific″ [14].

Along this vein, we seek to emphasize the importance of not only feminist, intersectional, and participatory methods for decolonizing global health work and research, but also how these methods, in combination with African feminist perspectives, can make an important contribution to decolonizing global health research and work in SSA, specifically. This paper draws on four interconnected methodological approaches and perspectives to address power imbalances in global health research. Feminist methods can be used to center the experiences of marginalized groups and disrupt patriarchal knowledge production systems that have historically excluded women and minoritized voices and perspectives. Intersectional analysis examines multiple systems of oppression, including race, gender, class, sexuality, and colonial histories, that interact to create marginalization that cannot be understood through other frameworks. Participatory research methods redistribute power in the research process by positioning community members as co‐researchers rather than subjects, fundamentally transforming who counts as an expert and what counts as knowledge. African feminist perspectives provide contextually grounded epistemologies and resistance to both patriarchal and colonial forms of power. While these approaches overlap, they also make specific analytical contributions. These perspectives and methods used together can provide a holistic framework for conducting collaborative, contextually grounded knowledge production in global health research that is beneficial to local communities in SSA.

## 2 Why Feminist Methods Matter for Global Health Research in SSA

Feminist theorists have highlighted the need to center knowledge production around the embodied realities and narratives of those typically “othered” or positioned at society's margins [17, 18, 19]. This epistemological shift is particularly crucial in SSA contexts where colonial and neocolonial research practices have systematically devalued local knowledge systems. Black feminist and critical race theorists in the U.S. have specifically identified the need to include marginalized voices while attending to intersectional or interlocking forms of oppression. An intersectional analysis attends to “…systems of race, social class, gender, sexuality, ethnicity, nation, and age” which create interlocking forms of oppression and discrimination [17, 20], examining multilevel (interpersonal, community, structural) experiences of marginalization [21].

This analytical framework proves essential for understanding how structural oppression operates across personal, interpersonal, community, societal, and historical/colonial levels in SSA contexts. Eger et al (2024) emphasize that properly understanding health inequities requires investigating the intricate interplay of power dynamics and structural determinants, including gender, socioeconomic status, and race [14]. Such understandings can enhance research design relevance, particularly in contexts where neoliberal structural adjustment and globalization affect local access to resources for specific populations.

These theoretical principles translate into transformative research practice, as demonstrated by the “Empowerment for Us by Us” study in Kisumu, Kenya. The study exemplified feminist methodology by positioning sexual and gender minority persons assigned female at birth as co‐researchers rather than subjects [22]. Rather than imposing definitions of empowerment, the study sought to identify research participants’ definitions according to their own cultural contexts and lived realities. This approach revealed how Western conceptualizations of individual empowerment can overpower local understandings—insights that would have been invisible through traditional survey methods.

Feminist epistemological perspectives prioritize diverse knowledge forms—narrative, storytelling, oral history, embodied experience—as legitimate science. Without careful consideration of these intersectional experiences through social categories such as sex, gender, race, and historical processes such as colonization, the biomedical positivist paradigm may reinforce unexamined assumptions about complex social phenomena [4]. Categories and approaches to understanding gender and sex can become particularly rigid in Euro‐centric, positivist paradigms, rather than being understood as socially constructed within specific cultural contexts [11, 23, 24, 25].

Feminist approaches offer “analytic weapons” to disrupt positivist logics, providing perspectives grounded in the diverse realities of individuals living and navigating complex contexts [26]. Methods such as ethnography, participant observation, participatory action learning, storytelling, interviews, focus groups, and photo‐voice can focus on critically important social contexts while confronting intersectional power dynamics [27]. Intersectional thinking in research methodologies enables data disaggregation to include various social factors when collecting data, with the goal of understanding across various such factors, extending identification options beyond gender and sexual binaries while developing culturally attuned measurement approaches [28].

## 3 Why Participatory Methods Matter for Global Health Research in SSA

Feminist participatory research combines both a feminist epistemological perspective, providing a method for centering research on the voices and lived experiences of often marginalized peoples [6, 16, 28, 29, 30, 31, 32]. While participatory research methods, like feminist methods, are grounded in the realities of marginalized communities, they also focus on collaborative, community‐centered, and policy‐driven results, which are informed by the specific needs of marginalized communities. In addition, participatory methods are grounded in methodological approaches that re‐situate who counts as an expert and what counts as knowledge, critically challenging power dynamics between the researcher and researched [33, 34]. Participatory methods include action research, participatory action research, and community‐based participatory research, which have influences from the social sciences [35]. Action research is defined as when communities identify their issues, plan, take action, and then evaluate the results [35]. Similarly, participatory action research and community‐based participatory research are centered on the idea of conducting research with members of marginalized groups, which includes co‐learning, sharing of expertise, shared decision making, and mutual ownership, with the goal of the research being to create social change [35, 36, 37]. Feminist participatory action research is a combination of feminist frameworks and theory and participatory action research methodologies, with the goal of forming community‐based engagement, partnership, and creating social change through policy and action [28, 31, 38, 39]. Feminist participatory action research methods can not only analyze power and inequity, but also create space for the development of political awareness [32]. These methodologies are not only critical for breaking down traditional scientific relationships in global health, but they are crucial for creating equitable, sustainable partnerships, relationships, and solutions for community health needs [29, 39, 40].

## 4 Why African Feminism Matters for Global Health Research in SSA

A multiplicity of forms of social movements have fueled policy, legal, and gender based reforms throughout the African continent [41, 42, 43]. African feminism has historically and commonly focused on economic justice and resistance to both colonial and neocolonial exploitation [44]. The complex, multi‐lingual, and multi‐ethnic context of post‐colonial SSA includes a multiplicity of African feminist perspectives that challenge the “global North‐South knowledge system” that can perpetuate neo‐colonial knowledge production [41]. In the context of global health research, African feminist perspectives can provide valuable insights into the dominant biomedical frame employed in the global health sector that invisibilizes various forms of knowledge. African feminist perspectives bring to the surface systemic issues such as colonial legacies, cultural biases, and economic inequities and, in turn, help researchers develop more contextually sustainable and relevant health initiatives, as well as solutions to gender inequities. This standpoint provides a perspective that is crucial in exploring unique challenges such as reproductive rights, access to healthcare, and inclusion in healthcare decision‐making.

In line with intersectional perspectives, African feminist perspectives also highlight inequitable social relations specific to SSA contexts [13, 14, 45]. Feminist scholar Sylvia Tamale urges the use of intersectionality as a method in qualitative research, through which interpretive narratives, life‐histories, and case studies″ can be developed and “in‐depth and rich data” through “thick descriptions” may offer specific information attuned to lived experiences [27]. African feminist perspectives have also called on researchers and scholars to challenge Euro‐centric perspectives and their transferability to different cultural settings. African feminist scholar Amina Mama notes that in the SSA context, Northern feminist conceptions of identity politics as individualistic may not map onto feminist issues of material redistribution and justice in the SSA context that are intertwined with an existential need for security and integrity [41].

African feminists have also highlighted the philosophy of Ubuntu as a critical framework. Ubuntu, translated as “I am because we are,” refers to the mutual interdependence of persons and the collective socio‐economic nature of communities that are central for survival in the SSA context [46, 47]. The concept can be a useful ethical framework, as well as a methodological approach for health research. Jessica Horn proposes that Ubuntu‐informed feminist perspectives can acknowledge the specificities of social and economic existence and interconnectedness in the SSA context [46]. An Ubuntu‐centered approach could prove helpful in informing HIV prevention and research. Current global health interventions often focus on individual behaviors, such as condom use and PrEP adherence. An Ubuntu‐centered approach would examine community networks that could help to bolster individuals' help through social support, peer education systems, and communal economic resources, moving towards community‐based solutions rather than individual change.

African feminist perspectives highlight how indigenous knowledge systems can offer more appropriate and holistic approaches to health and wellbeing that have been historically erased and devalued in colonialism and global health research. Udenigwe (2024) explains that in a maternal health research project in Nigeria, an outsider researcher predicted a gender framework that inaccurately attributed decision‐making to men as leaders of their households [48]. This framework neglected the important role played by elderly women and mother figures in child health issues, and the concept of access to power through age hierarchies [48]. Similarly, African feminist writings focusing on the Igbo and Yoruba tribes of Nigeria show that older women played an important role in safeguarding maternal health in informal contexts and communities in general through rules that protected women and children [49]. These findings demonstrate the significance and importance of engaging directly with indigenous knowledge and frameworks in order to develop appropriate and effective health interventions that can combat the existing health challenges.

In summary, African feminist perspectives and approaches can challenge extractive research practices in global health research. Such perspectives challenge predetermined agendas and ground research in methodologies that are centered on community‐based knowledge and solutions. Such an approach can include collaboratively defining problems through community‐based frameworks and concepts, incorporating indigenous research methods such as dialogue, oral history, and traditional practices, and using community‐based analysis that reflects Ubuntu‐focused problem‐solving.

## 5 Conclusion

This paper has explored how global health research remains embedded in colonial histories of power to rely on biomedical and positivist logics that are unable to capture the complexity of health challenges in SSA. The use of feminist, intersectional, and participatory methods, and African feminist perspectives, can help to provide potential decolonizing approaches to global health research and interventions. The use of these methods and perspectives in the SSA context not only challenges “traditional” research relationships but is also driven by the knowledge of communities’ experiences of health disparities, and leads to context‐specific or localized solutions to complex health inequities.

African feminist offerings such as Ubuntu provide frameworks for reimagining health research and identifying community‐based solutions that can challenge structural forms that are at the root of health inequities. Research that is grounded in local knowledge systems, that builds community research capacity and focuses on long‐term sustainable relationships and solutions, challenges extractive research models, and leads to long‐term change.

The implications of the use of these methods and frameworks offer transformative potential for global health research practice. However, for change to take place, substantial material shifts are necessary. This includes a willingness of donors to fund the use of specific methodological approaches, including the provision of sustained, long‐term funding to support community‐engaged research that includes capacity building, and provides direct funding to African‐based institutions and researchers. Global health education approaches must include African feminist, indigenous, intersectional, and participatory methods training as a part of a central curriculum. The understanding of local history, context, and knowledge should be made a requirement for any researcher working in SSA. Ethical frameworks in research must be extended to include who owns and controls the process of knowledge production.

The use of inaccurate and inappropriate research methodologies and approaches in global health is not just an academic limitation but a substantial ethical failure. The use of feminist, intersectional, participatory, and African feminist perspectives and methods provides a pathway towards genuine health equity in SSA. Such approaches provide global health researchers with solutions embedded in collaboration, mutual respect, and African‐based expertise, providing a departure from a colonial past, towards effective and meaningful health interventions grounded in local knowledge‐based solutions.

## Conflicts of Interest

The authors declare no conflicts of interest.

## Data Availability

The authors have nothing to report.
